# Support Needs and Interventions for Family Caregivers of Patients with Amyotrophic Lateral Sclerosis (ALS): A Narrative Review with Report of Telemedicine Experiences at the Time of COVID-19 Pandemic

**DOI:** 10.3390/brainsci12010049

**Published:** 2021-12-30

**Authors:** Giulia D’Alvano, Daniela Buonanno, Carla Passaniti, Manuela De Stefano, Luigi Lavorgna, Gioacchino Tedeschi, Mattia Siciliano, Francesca Trojsi

**Affiliations:** Department of Advanced Medical and Surgical Sciences, Università degli Studi della Campania “Luigi Vanvitelli”, 80138 Naples, Italy; giulia.dalvano@studenti.unicampania.it (G.D.); daniela.buonanno@policliniconapoli.it (D.B.); carla.passaniti@unicampania.it (C.P.); manuela.destefano@policliniconapoli.it (M.D.S.); luigi.lavorgna@policliniconapoli.it (L.L.); gioacchino.tedeschi@unicampania.it (G.T.); mattia.siciliano@unicampania.it (M.S.)

**Keywords:** amyotrophic lateral sclerosis, caregiver, support, telemedicine, COVID-19

## Abstract

Family caregivers of people with amyotrophic lateral sclerosis (ALS), a severely disabling neurodegenerative disease due to the degeneration of both upper and lower motor neurons, have a very demanding role in managing their relatives, thereby often experiencing heavy care burden. Previous literature has widely highlighted that this situation reduces caregivers’ quality of life and increases their psychological distress and risk of health problems, but there are relatively few studies that focus on psychological interventions for these situations. Family support is more—not less—important during crisis. However, during the COVID-19 pandemic, maintaining public safety has required restricting the physical presence of families for hospitalized patients. Caregivers of ALS patients felt increased sense of loneliness and experienced greater difficulties in the access to both hospital and home assistance. In response, health systems rapidly adapted family-centric procedures and tools to circumvent restrictions on physical presence. In this regard, internet-based and telehealth solutions have been adopted to facilitate the routine, predictable, and structured communication, crucial to family-centered care. This narrative review aims at addressing more current matters on support needs and interventions for improving wellbeing of caregivers of ALS patients. In particular, we aimed at highlighting several gaps related to the complex needs of caregivers of ALS patients, to the interventions carried out in order to respond to these needs, and to the changes that COVID-19 pandemic caused from 2020 to nowadays in clinical managing of ALS patients. Finally, we report ongoing experiences of psychological support for family caregivers of ALS patients through telehealth solutions, which have been reinforced in case of needing of physical distancing during the COVID-19 pandemic.

## 1. Introduction

Amyotrophic lateral sclerosis (ALS) is a progressive neurodegenerative disease due to the degeneration of both upper and lower motor neurons. Its clinical presentation is heterogeneous with symptoms comprising muscle weakness, spasticity, dysarthria, dysphagia, and respiratory failure [1]. In addition, up to 50% of ALS patients may develop cognitive and/or behavioral impairment during the course of the disease, mainly represented by executive and language dysfunctions, among cognitive alterations; and apathy and disinhibition, among behavioral disturbances [2,3]. Since there is no curative treatment, the best approach for improving disease management and the patients’ quality of life is the implementation of multidisciplinary and palliative care [4,5]. Moreover, due to the heterogeneous phenotypes of ALS, interventions should be individualized based on “different contexts and unique situations” [6]. As the disease progresses, patients with ALS experience a loss of autonomy in daily life activities, becoming more dependent on their caregivers. It is not uncommon that relatives assume primary responsibility for the complex care needs of patients in non-medicalized setting and, in this regard, ALS has been described as a “family illness” [7].

**Figure 1:**
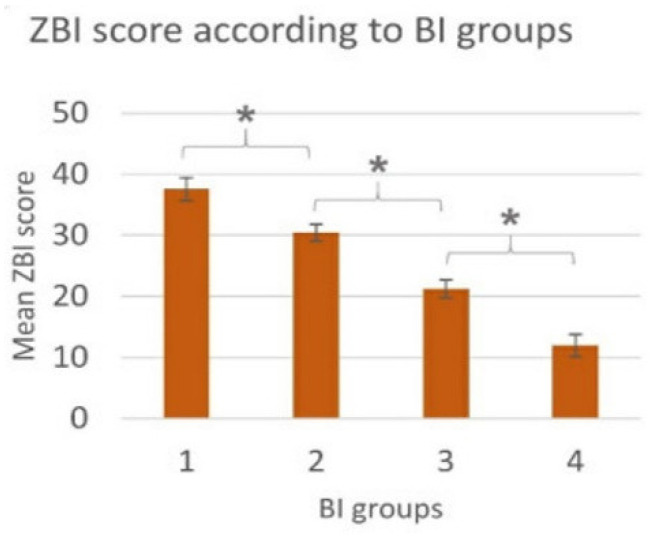
Schischlevskij et al. [21] revealed statistically significant differences in ZBI in subsets of caregivers of ALS patients classified according to functional status of patients (i.e., assessed using Barthel Index, BI—4 ascending stages of disease severity): ZBI-BI groups (ANOVA: *p* < 0.001; KWT: *p* < 0.001; *r*_s_ = −0.542, *p* < 0.001, *n* = 242), ZBI-BI (*r*_p_ = −0.555, *p* < 0.001, *n* = 242); * = post-hoc: *p* ≤ 0.05 between subgroups. Derived from Schischlevskij et al. [21], open access article distributed under the terms and conditions of the Creative Commons Attribution (CC BY) license (http://creativecommons.org/licenses/by/4.0/ accessed on 29 December 2021).

**Figure 2:**
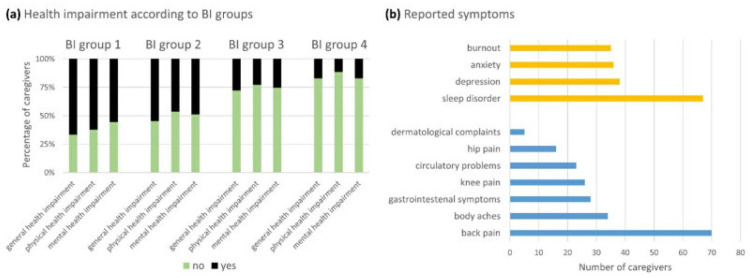
Schischlevskij et al. [21] addressed caregivers’ health impairment. (**a**) Three adjacent columns were assigned to each BI group (groups 1–4). The extent of the colored sections represents the percentage of caregivers. A statistically significant increase in the incidence of physical and mental health impairment was observed according to the BI group (i.e., BI group 4 = full independence; BI group 1 = full dependence). (**b**) Physical and mental symptoms were reported. Derived from Schischlevskij et al. [21], open access article distributed under the terms and conditions of the Creative Commons Attribution (CC BY) license (http://creativecommons.org/licenses/by/4.0/ accessed on 29 December 2021).

Informal caregivers represent a physical and emotional support to patients and have a key role in the process of decision-making. However, the role of caregiver can be a very demanding and overwhelming activity for relatives [8,9], who often experience heavy care burden [10,11,12,13] and may develop somatic symptoms [14] and psychological distress [15,16], needing to implement coping strategies to remove, reduce, or manage stressful situations [17,18,19,20]. For instance, recently, Schischlevskij et al. [21] revealed that ALS caregivers’ burden, recorded by the Zarit Burden Interview (ZBI), had mean ZBI = 26/88 (i.e., with regard to ZBI score, 0 = no burden, ≥24 = highly burdened) and this score was correlated with patients’ functional status (*r*_p_ = −0.555, *p* < 0.001; *n* = 242) (Figure 1). Moreover, longer duration of caregiving (median hours per day, 3 h/day), as well as having less social support, was associated with individual health deterioration due to caregiving in 42.6% of informal caregivers [21]. Higher depression and anxiety scores (i.e., median score of Hospital Anxiety and Depression Scale-depression subscale -HADS-D- 8/21 and Hospital Anxiety and Depression Scale-anxiety subscale -HADS-A- 9/21) and lower health-related quality of life scores (i.e., mean EuroQol Five Dimension Five Level Scale index value 0.845/1) of ALS caregivers were related to the patients’ functional status, which was revealed to heavily influence caregivers’ burden (Figure 2). Therefore, the caregiving strain has been shown to result from complex interactions between patients’ functional status and caregivers’ own mental health and demographic aspects [21]. In particular, patients’ functional status, such as wheelchair dependency, and caregivers’ own mental health issues, female gender, and younger age were associated with higher burden [16,21,22]. Caregivers are more frequently represented by partners and, among those, women tend to report higher burden [21,22] and anxiety (i.e., female CGs with a median HADS-A score of 9/21 showed the presence of anxiety [21]). Moreover, partners scored higher in several dimensions of the caregiver burden when compared to sons and daughters [22].

Supportive interventions, aimed at reducing psychological distress and focused on reducing utilization of maladaptive coping strategies, have been developed in the last decade for improving wellbeing of caregivers of ALS patients [23,24,25,26,27,28,29,30,31,32], which in turn may even result in a better disease management.

Taking into account that using e-health may help tailor interventions to the caregivers’ support needs [33], telemedicine approaches have been adapted to remotely manage ALS patients improving the efficiency of healthcare [34,35]. In particular, due to Coronavirus disease 2019 (COVID-19) pandemic, containment measures led to necessary restrictions in personal movement and reduced access to traditional healthcare resources. Healthcare systems faced a complete rearrangement of resources and spaces; most visits and interventions for chronic diseases were cancelled, postponed, or converted to telemedicine [36]. Bombaci et al. (2021) addressed telehealth approaches aimed at remotely assessing neurological status, global disability scores, such as the revised ALS Functioning Rating Scale (ALSFRS-R) [37], respiratory and nutritional functions and at remotely providing psychological support for both patients and caregivers [38].

The purpose of this narrative review is to address more current matters on support needs and interventions aimed at improving wellbeing of caregivers of ALS patients. In particular, we aimed at highlighting several gaps related to: (1) the complex, partially unmet needs of caregivers of ALS patients; (2) the interventions carried out in order to meet these needs; and (3) the changes that COVID-19 pandemic caused from 2020 to the present in clinical managing of ALS patients, reinforcing the role of telemedicine to remotely support ALS patients and their caregivers.

## 2. Literature Search

We searched literature from 2015 to June 2021 in PubMed, the most popular hub of updated bibliographic information on scholarly biomedical journals and online books, using the search terms “amyotrophic lateral sclerosis” or “motor neuron disease” in combination with “caregiver(s)”, “burden”, “support”, and “COVID-19”. To note, since COVID-19 pandemic limited people movements and their access to healthcare services from 2020, telemedicine emerged as a growing tool to deliver health assistance remotely to chronic patients, including psychological support. Consequently, we also used the search terms “telemedicine” or “telehealth” in combination with the above-mentioned ones. Only studies in English language and papers from peer-reviewed journals were included. We excluded editorials and case reports. Letters to the editor were included when reported original results in brief. Systematic/scoping/rapid reviews and metanalysis were also included. The final reference list was generated on the basis of originality and relevance to the topics covered in this manuscript. Emphasis was placed on publications from the last 5 years, but we did not exclude highly regarded older publications. Moreover, we described ongoing telehealth services for ALS patients and their family caregivers during the COVID-19 pandemic.

Among 215 references derived from the search, 41 manuscripts were selected according to the above-mentioned criteria. Several papers (*n* = 15) were cited in the following sections, although most papers (*n* = 26) have been cited in the introduction [7,8,9,10,13,14,15,16,18,19,20,21,22,23,24,25,26,27,28,29,30,31,32,34,35,38]. In all included studies, caregivers were patients’ family members. Moreover, all included studies mentioned several needs of ALS patients’ caregivers and, among those, 14 manuscripts presented potential psychological approaches to them. The main findings were synthesized in the following sections. The order of presentation was not hierarchical.

## 3. ALS: Family Caregiver Needs

The wellbeing of caregivers is crucial for a careful management of chronic diseases: a high level of burden may lead to a breakdown in care and to an earlier placement of the patient in a care home or hospice [39]. Among the most mentioned needs of caregivers there are practical, social, informational, psychological, physical, emotional, and spiritual ones, categorized into Fitch’s Supportive Care Needs Framework (SCNF) [40], widely used for cancer care and other various chronic conditions [41,42] and used for ALS through the validation of the ALS supportive care needs (ALSSCN) assessment [43,44]. Practical needs can vary from help with assisting devices [26] to help in managing the logistics of care provision and applying for resources, which is often time consuming, bureaucratic, and a source of stress [23].

With regard to informational needs, it is common for both patients and caregivers to need education on ALS management, in terms of the disease itself and about caring for a person with ALS [45]. Similarly, many caregivers have expressed the necessity of more personal time in some studies [23]. Most relatives who are informal caregivers adapt their lifestyle to that of the patients and have revealed to neglect their own needs but focus on the patient [26]. In a study performed by de Wit et al. [23], the enrolled caregivers (n= 15; 7 wives, 4 husbands, and 4 daughters) indicated that being able to leave the house and spend time on their own activities was important for maintaining their own wellbeing and allowed them to divert their attention from ALS. On the other hand, they experienced difficulties in handing over care to others, which often caused distress rather than relief. Additionally, when they used paid home-care, they were confronted with reduced privacy [23].

Importantly, caregiver needs are dynamic in ALS in that they change as the disease progresses. In this regard, Williams et al. [18] revealed that the trajectory of the ALS family caregivers’ needs can be represented by a four-cluster map: (1) early coping and adjustment; (2) maintenance; (3) transition to end stage; and (4) coping with change and loss. Moreover, caregivers who resided with their ALS family member showed poorer mental and physical health than family caregivers who did not have primary residence with the ALS patient [18].

Galvin et al. [46] also described a changing in caregivers’ needs, depending on ALS patients needs and on their abilities of meeting them. Remarkably, an additional source of stress was related to uncooperative behaviors of patients and resistance to external support and services (i.e., over-reliance on the informal caregiver, refusal of services, or unwillingness to engage when service providers were present). In particular, previous evidence revealed that compliance with non-invasive ventilation is adversely affected by cognitive impairment [47,48] and that behavioral deficits in ALS have a significant negative impact on caregivers’ quality of life increasing carer burden considerably [9].

As for the coping strategies used by caregivers, spirituality can sustain hope and diminish despair [45]. Siciliano et al. [19] have examined the association between coping strategies and psychological distress in 96 caregivers of ALS patients. They found out that ALS caregivers who adopted the emotion-oriented coping strategy were those with higher levels of psychological distress compared to those who adopted task-oriented strategies. Moreover, a significant relationship of patients’ functional dependence levels with burden experienced by caregivers was observed. These results may support the effects of coping strategies on intensity of burden, depression and anxiety related to the role of caregiving. Therefore, interventions aimed at reducing utilization of maladaptive coping strategies may improve wellbeing in ALS caregivers [19].

## 4. Support Interventions for Family Caregivers of Patients with ALS

Despite the need for specific psychological interventions for caregivers, there are relatively few studies addressing this topic. Recently, de Wit and colleagues [24,25] proposed for the first time a blended intervention (face-to-face contact and e-health) based on Acceptance and Commitment Therapy (ACT), a cognitive behavioral therapy that encourages individuals to accept events that are out of their control and to identify important values in life in order to engage in committed action to pursue these values [49]. Participants were partners of ALS or progressive muscular atrophy (PMA) patients: 74 caregivers started with the support program and 74 caregivers were on a wait list for the support program. The majority of caregivers were women (64.9%); their mean age was 61 years. A total of 28 (19%) caregivers dropped out of the study, the most common reason for drop-out being the death of the patient. Moreover, dropouts had partners with a significantly lower physical functioning score compared to study completers. The caregivers undergoing to the support program referred that it helped them to be more aware about their own situation and to perceive more control over it, empowering caregivers to make choices according to their own needs. Moreover, the ‘supported’ caregivers reported increased attention to the relationship with their partner and felt acknowledged. The online approach was also appreciated: caregivers may experience a lack of personal time, since they spend many hours on providing care, especially in the advanced stages of ALS. Using the online support enabled them to enter the program at their preferred time and place. Conversely, mindfulness and peer contact intervention was not always appreciated, indicating that support intervention should be personalized, with the possibility to choose options [24]. With regard to caregivers who completed the intervention (38 caregivers completed the program in an average of 8 weeks and spent a mean of 1 h and 26 min per module), the support program had no effect on their psychological distress, burden, quality of life, and patients’ quality of life and psychological distress, although significant positive effect was revealed on caregivers’ feeling of control over caregiving (measured by the Revised Scale for Caregiving Self-Efficacy subscale controlling -RSCSE-Contr; *p* = 0.007) [25]. Of note, almost half of the caregivers did not complete the intervention with the most frequently reported reason being lack of time and, therefore, the high level of dropouts may have limited the ability to detect an intervention effect [25].

Based on caregivers’ need for peer contact, group interventions were also developed [28]. Mutual support groups have been described by participants as helpful experiences: having shared experiences allowed participants to feel less alone, better understood, more accepting of their beloved one, more trusting of each other and helped them to improve their self-esteem and self-efficacy. Moreover, they improved their coping strategies. However, when the group is too heterogeneous and includes some participants who show hostility, this may hinder the integration of this minority [28].

Ugalde et al. [31] also reported positive feedback from 13 caregivers participated in a group intervention session which was focused on self-care, problem-solving, and mindfulness, although there was no significant change in measures between pre-intervention and 6 weeks post intervention. Recently, Burke et al. [30] performed a randomized controlled trial, comparing two intervention groups, undergone, respectively, mindfulness-based stress reduction (MBSR) and cognitive behavioral therapy (CBT), to a wait list control (treatment as usual) group from a database of 75 caregivers of ALS patients. MBSR has been shown to promote the ability to cope with the management of negative emotions, to mitigate the effects of disease burden and to promote psychological adaptation in caregivers of ALS patients [50], while CBT interventions have the most extensive evidence base for treating anxiety and depression during short-term intervention blocks [51].

## 5. Outbreak of COVID-19 Pandemic: Telemedicine Services for ALS Patients and Caregivers

In the last year, the breakout of COVID-19 pandemic has confined the majority of the world population to their homes. In this scenario, people were invited to seek medical help only in acute situations, to prevent the spread of the infection. Consequently, the follow-up of patients with chronic disease, such as ALS patients, was suspended through in-person visits and many ALS centers moved to telemedicine in order to continue to give assistance to patients and their caregivers [52,53,54,55]. Multidisciplinary tele-consults have been also implemented before COVID-19 pandemic. Telehealth monitoring conducted in two Australian hospitals highlighted the feasibility of video consults, even if respiratory and palliative concerns arose [56]. In particular, 38 patients were monitored, an average of three times at 3–4-monthly intervals, for about 12 months beyond the last tertiary hospital visits [56]. Furthermore, Geronimo et al. [57] assessed the feasibility and acceptability of telehealth for ALS care via real-time videoconferencing from the clinic to patients’ homes. To this scope, participating patients (*n* = 11), caregivers (*n* = 12), and health care providers (*n* = 15) completed surveys assessing satisfaction with the visit, quality of care, and confidence with the interface, reporting high levels of satisfaction from ALS patients, their caregivers and most multidisciplinary team members. The reported benefits included continuing providing care, understanding home dynamics of patients/caregivers dynamics, no travel requirements, time savings in addition to less fatigue and stress for the patients [57]. Hobson and colleagues [58] collected information about the condition of patients and caregivers through questionnaires on an Android app (Tele-health in Motor Neuron Disease, TiM) on a weekly basis. They reported various potential benefits: improved communication and care coordination, reassurance, identification of complications, and being an alternative or addition to clinics [58].

During the COVID-19 pandemic, the use of telemedicine in the care of ALS expanded. In Turin, Vasta, and colleagues [55] performed remotely 139 visits between March and April 2020, mostly via phone calls, reserving videoconferencing to psychological and speech therapy consults. During the tele-visits, patients were asked about general conditions, specific ALS symptoms and signs, and most common COVID-19 symptomatology. The ALSFRS-R was also collected [55]. Similarly, Capozzo et al. [52] conducted remote visits for 32 ALS patients, collecting clinical information through a specific questionnaire aimed at identifying significant changes or problems since the last evaluation, with the objective of evaluating whether multidisciplinary assessment of ALS patients using telemedicine was feasible and acceptable to patients and caregivers. All patients enrolled in their study refused video interaction, mostly because of the lack of a computer or a smartphone [52]. On the contrary, the cohort studied by De Marchi et al. [53] preferred video-calling, as it gave the perception of talking face-to-face to healthcare professionals. In this study, authors provided multidisciplinary visits—involving a neurologist, a dietician, a psychologist, and a physiotherapist—through an online platform (IoMT Connected Care Platform (Ticuro Reply) [53].

Telemedicine demonstrated to be effective in ALS patients’ care. However, its approaches have pros and cons. Among the reported advantages, telemedicine was able to identify potential critical situations and to prescribe aids [53]. Moreover, it avoided disadvantages related to travel to ALS centers with the consequent reduction of time and economic costs [52]. Substantially, patients and their caregivers have been shown satisfaction with telemedicine approaches [52,55]. However, most of them referred to the lack of in-person visits as cons [55], probably due to the unique patient–doctor relationship that it allows. Globally, telemedicine helped patients and caregivers to not feel abandoned, especially in case of needing of physical distancing, such as during COVID-19 pandemic. Social isolation and loneliness are among the most important adverse consequences of the pandemic in ALS patients and their caregivers. In fact, among the main source of anxiety during COVID-19 pandemic, there were feelings of being forgotten/rejected by clinicians [59].

With regard to ALS caregivers, most of them felt their burden increased after the imposition of national quarantine during COVID-19 pandemic, especially in case of co-occurrence of cognitive/behavioral impairment with ALS. In particular, a study conducted on a cohort of 30 ALS patients and 29 caregivers, who compiled questionnaires assessing pandemic distress, mood, loneliness, burden, and behavioral changes, showed that in the caregivers’ group the main sign of pandemic distress was a change in the degree of anxiety [59]. Surprisingly, authors did not find significant association between worries of getting COVID-19 and functional disease severity, stage, or clinical phenotypes [59]. Besides limiting the access to healthcare, national quarantines restricted the possibility of meeting friends or family members and of receiving home assistance. Consequently, caregivers perceived lower family help and worsening of homecare assistance [60]. On the other hand, ALS patients took advantage of the time spent at home to perform new activities with their families [60].

## 6. Limits

Our work has several limitations. First, the literature search was performed in a single database and this may have led to potential omissions of contributing sections of the literature. Second, the examined temporal range refers mainly to the last five years. Third, narrative review, as a typology of review, has intrinsic limitations, in that it may lack an explicit intent to maximize scope or analyze data collected in comparison to systematic review [61].

## 7. Conclusions and Future Perspectives

Needs of ALS patients’ caregivers include practical, social, informational, psychological, physical, emotional, and spiritual issues, being dynamic during the ALS course in that they change as the disease progresses. Thus, in the last decade supportive interventions in response to these needs, aimed at reducing psychological distress and utilization of maladaptive coping strategies, have been developed for improving wellbeing of caregivers of ALS patients (Table 1).

In this scenario, more recently, COVID-19 pandemic confined the majority of the worldwide population to their homes and many ALS centers moved to telemedicine services for continuing to give assistance and support to ALS patients and their caregivers. Substantially, patients and caregivers were satisfied with telemedicine approaches, although some of them would have preferred in-person visits, probably due to the unique patient–doctor relationship that it allows (Table 1). The COVID-19 outbreak prompted a wider use of telemedicine services, suggesting that telemedicine for ALS patients should be intended to be complementary to in-person care and only in some cases to replace it depending on circumstances and patient/carer preferences.

Future longitudinal analyses should be extended to larger samples of caregivers of ALS patients. In-person and/or remote psychological support interventions should be designed using individual and/or group-based approaches with the aim of reducing caregivers’ burden and loneliness and increasing their resilience.

## Figures and Tables

**Table 1:** Types, benefits, and limits of support interventions to caregivers of ALS patients before and after COVID-19 pandemic occurrence.

Caregivers’ Support before COVID-19 Pandemic Occurrence				
References	Type of Intervention	In-Person vs. E-Health	Benefits	Limits
[25] de Wit et al. (2020)	Acceptance and Commitment Therapy (ACT), including psychoeducation, psychological and mindfulness exercises, practical tips and information, contact with peers and counsellors	Blended (in-person and e-health)	Increasing feeling of control over the caregiving situation; acceptance of negative emotions and thoughts; increased attention to partner relationship.	Mindfulness exercises and peer support were not always appreciated.
[28] Cipolletta et al. (2018)	Mutual support groups	In-person	Overcoming isolation; improvement in emotional and mental states; better coping with ALS and its consequences; improvements in care for their relatives.	Lack of regularity in participation; too much time criticizing the healthcare system.
[31] Ugalde et al. (2018)	Group intervention focused on self-care (for the caregiver), problem-solving and mindfulness	In-person	High appreciation of the group format.	No improvement in distress measures
[50] Pagnini et al. (2016)	Online caregiver survey to assess mindfulness, burden, quality of life, anxiety, and depression	E-health	Mindfulness was negatively correlated with burden, depression, and anxiety, and was positively correlated with quality of life.	
Caregivers’ support after COVID-19 pandemic occurrence				
References	Type of intervention	In-person vs. e-health	Main findings	
[59] Consonni et al. (2021)	Online questionnaires assessing pandemic distress, mood, loneliness, caregiver burden (CBI), and behavioral changes	E-health	Increasing levels of anxiety, probably due to social isolation and loneliness	
[60] Giusiano et al. (2021)	Administration of phone scales assessing patients’ Quality of Life (QoL), general health status (EQ-5D-5L) and caregivers’ burden (Zarit Burden Interview), during lockdown (T1) and 1 month after it ended (T2)	E-health	T1: patients’ QoL was inversely related to caregivers’ burden. Caregivers felt abandoned.	
